# Cotton stalk-derived hydrothermal carbon for methylene blue dye removal: investigation of the raw material plant tissues

**DOI:** 10.1186/s40643-021-00364-8

**Published:** 2021-01-30

**Authors:** Libo Zhang, Junyan Tan, Gangying Xing, Xintong Dou, Xuqiang Guo

**Affiliations:** 1 State Key Laboratory of Heavy Oil Processing, College of Engineering, China University of Petroleum-Beijing at Karamay, Karamay, 834000 People’s Republic of China; 2 Shenzhen College of International Education, Shenzhen, 518048 People’s Republic of China; 3 State Key Laboratory of Heavy Oil Processing, China University of Petroleum, Beijing, 102249 People’s Republic of China

**Keywords:** Cotton stalk, Acid/base pretreatment, Hydrothermal carbonization, Plant tissues, MB removal

## Abstract

Conversion of the abundant agricultural residual cotton stalk (CS) into useful chemicals or functional materials could alleviate the fossil fuels caused energy shortages and environmental crises. Although some advances have been achieved, less attention has been paid to the plant tissues effect. In this study, the plant tissue of CS was changed by part degradation of some components (hemicelluloses and lignin, for example) with the aid of acid/base (or both). The pretreated CS was transformed into hydrochar by hydrothermal carbonization (HTC) method. Morphological and chemical compositions of CS hydrochar were analyzed by various techniques, including elemental analysis, Fourier transform infrared (FTIR), BET analysis, X-ray photoelectron spectroscopy (XPS) and X-ray diffraction (XRD). Methylene blue (MB) removal of prepared CS hydrochar was used to evaluate CS hydrochar pollutions adsorption capacity. Results reveal acid/base (or both) pretreatment is beneficial for CS raw material to prepare high-quality CS hydrochar. The effects of some parameters, such as initial MB concentration, temperature, pH value and recyclability on the adsorption of MB onto both acid and base-pretreated CS hydrochar (CS-H_2_SO_4_ + NaOH-HTC) were studied. The present work exhibits the importance of agricultural waste biomass material plant tissues on its derived materials, which will have a positive effect on the direct utilization of waste biomass.
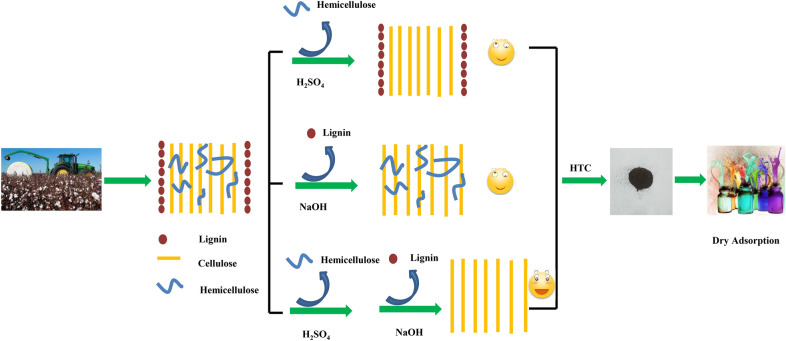

## Background

Over-reliance on fossil fuels has caused energy shortages and environmental crises. Looking for sustainable and inexpensive resources that can be obtained via environmentally friendly routes has never stopped. Conversion of non-food biomass into useful chemicals or functional materials is of great value on the sustainable development. Among those non-food biomass materials, cotton stalk (CS) is of large quantity. It is estimated that cotton production is about 25 million tons along with accounting for 50 million tons of biomass waste residues (Hamawand et al. 2016). Those residues mainly consist of CS, which is produced after cotton harvesting (Song et al. 2020). Usual treatments of CS include discard and burning that result in biomass material waste, environmental pollution and global warming issues (Foong et al. 2020; Medronho and Lindman 2015). Instead, developing conversion technology of CS into fuels, chemicals or functional materials could realize the recycle of waste agriculture residues and reduce incineration-caused environmental pollution. Besides, the high-value of CS can bring economic benefits that return raise rural incomes and further promote the cotton planting industry (Hamawand et al. 2016).

**Fig. 1:**
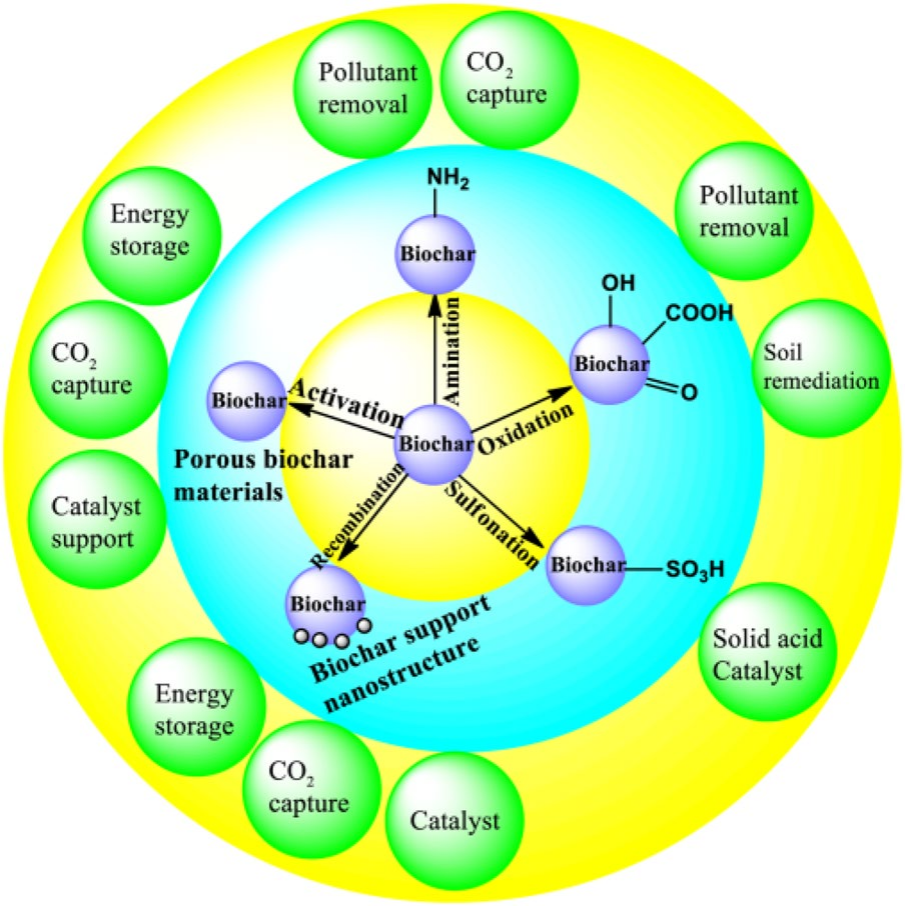
Demonstration of biochar as a platform carbon material for the synthesis of functional materials and their potential applications (Liu et al. 2015)

Up to the present, great progress has been made for the conversion of CS into useful fuels, chemicals and functional materials. Such as solar pyrolysis for bio-fuel production (Xie et al. 2019), co-pyrolysis with sewage sludge to use as soil amendments (Wang et al. 2020), hydrolysis for hydrogen generation (Li et al. 2020b), fermentation for ethanol production (Malik et al. 2020), synthesis of bio-degradable composites (Zhou et al. 2019), and carbonization for heavy metal ions removal (Hussain et al. 2020). Among those transformation technologies, carbonization has attracted tremendous attention for it is based on full component utilization of biomass raw materials, which is thus of economical efficiency (Liu et al. 2015; Ukanwa et al. 2019; Yang et al. 2019a). The produced carbonaceous material could be further used as a platform carbon material for the synthesis of various functional materials or directly used in some fields (as shown in Fig. 1) (Boostani et al. 2019; Liu et al. 2019; Vahdati-Khajeh et al. 2019; Yang et al. 2019b).

A common carbonization way for biomass materials is pyrolysis, including slow pyrolysis, fast pyrolysis, flash pyrolysis and pyrolytic gasification, which corresponds to biochar yield of 35–50%, 15–35%, 10–20% and 10–20% (wt%), respectively (Bridgwater. 2012; Chu et al. 2013; Laird et al. 2009). Although pyrolysis could obtain a high carbon yield with rich functional groups, the temperature is high [generally 600–900 °C (Guo et al. 2020)]. As an alternative carbonization way, hydrothermal carbonation (HTC) has significant advantages such as low operating temperature (180–250 °C) and the absence of a drying step, which reduces the energy consumption, and therefore, the operation cost is lower (Nizamuddin et al. 2017). To date, more attentions have been devoted to the regulations of hydrothermal conditions (temperature, retain time, atmosphere, catalysts, etc.), limited research focus on the feedstock plant tissues of biomass resource (Nizamuddin et al. 2017). Lignocellulosic biomass feedstock is composed of three major constituents: cellulose, hemicelluloses and lignin, which form a structure of cellulose microfibrils incorporated in a matrix of hemicelluloses and lignin in the plant cell wall (Machado et al. 2016; Zhang et al. 2018b), while the interaction among these three major constituents during the HTC process is not absolutely comprehended.

Due to the above facts, simple mineral acid/base (or both acid and base) pretreatment were adopted to selectively remove part of the CS composition (hemicelluloses or lignin) in this study. The pretreated CS subsequently went through the HTC and activation processes. Finally, the acid/base (or both) pretreated CS hydrochar was used as adsorbent to evaluate their methylene blue (MB) dye adsorption capacity. The influence of simple acid/base pretreatment was investigated to help understand the plant tissue effects on biomass derived materials from HTC process. Meanwhile, a simple acid/base pretreatment way was developed to prepare high MB adsorption capacity hydrochar from waste CS. The innovation of this work is to compare the major constituents and their effects during biomass HTC process for preparing high-quality hydrochar, which will provide some detailed understanding on biomass HTC process.

## Materials and methods

### Raw material and chemicals

CS was collected from local cotton fields (Karamay, Xinjiang, China) and pulverized into powder. Before the HTC process, the drying of grounded CS power was done for exact quantification. Sulfuric acid (H_2_SO_4_), sodium hydroxide (NaOH), potassium hydroxide (KOH), ethanol and hydrochloric acid (HCl) were supplied by the local supplier. Methylene blue (MB, C_16_H_18_ClN_3_S), glucose, cellulose, aniline and malachite green oxalate salt were purchased from Sigma-Aldrich. Deionized water (DI water, 18.25 MΩ cm) used in this study was prepared from a water purification system (Water Purifier, WP-UP-UV-20, Sichuan Water Technology Development Co. Ltd., China).

All chemicals used in this study were of analytical grade and used without further purification.

### Acid/base (or both) pretreatment of CS

**Fig. 2:**
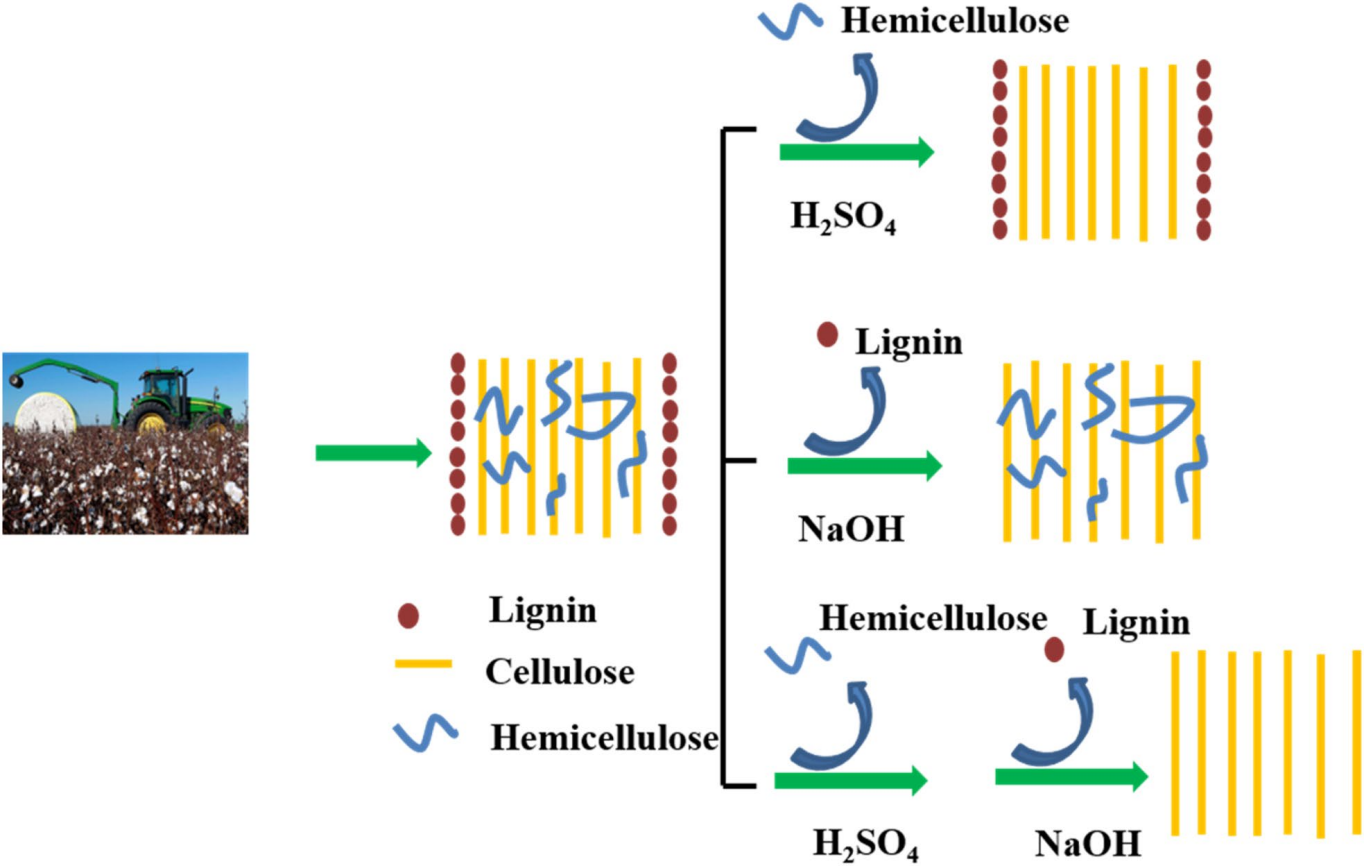
Diagram of influence of acid and base (or both) pretreatment on cotton stalk structure

CS powder was added into an H_2_SO_4_ (4%, wt%) or NaOH (5%, wt%) solution in water bath under vigorous stir at 95 °C for 4 h with a solid–liquid weight ration of 1:10. After pretreatment, the obtained solid was filtrated under reduced pressure and followed by drying in an oven at 105 °C till constant weight. Those solid were labeled as CS-H_2_SO_4_, CS-NaOH and CS-H_2_SO_4_ + NaOH (CS-H_2_SO_4_ + NaOH indicates CS underwent consecutive H_2_SO_4_ and NaOH pretreatment). The effects of H_2_SO_4_ and NaOH on CS tissue structure are illustrated in Fig. 2 (Mukherjee et al. 2017; Yu et al. 2019; Zhang et al. 2019).

### Hydrothermal carbonization and activation

A certain amount of CS-H_2_SO_4_, CS-NaOH and CS-H_2_SO_4_ + NaOH were mixed with DI water in a Teflon-lined autoclave (Zhang et al. 2020b) (Nantong Bo Run Petroleum Science) with a solid/liquid weight ration of 1:10. The autoclave was heated to 220 °C with a magnetic stir bar at 200 r/min and kept for 6 h. After cooling down to room temperature with the aid of flow water, the solid-state samples were filtered and washed thoroughly with DI water until the filtrate was neutral. The neutral solid-state samples were dried in an oven at 105 °C overnight.

Activation was performed in KOH solution at a weight ratio of 1:2 with excess amount of DI water, refluxed at 70 °C for 12 h (Salimi et al. 2017). The obtained power carbonaceous materials were washed thoroughly with DI water and dried in an oven. The samples were denoted as CS-H_2_SO_4_-HTC, CS-NaOH-HTC, CS-H_2_SO_4_ + NaOH-HTC.

Glucose, cellulose and crude CS also underwent the same HTC process and marked as Glucose-HTC, Cellulose-HTC and CS-HTC to further elucidate the role of CS plant tissue.

### CS-based HTC characterization

Elemental analysis of sample for carbon, oxygen, hydrogen and nitrogen was performed with an elementary analyzer (Flash EA 1112). Fourier transform infrared (FTIR) spectroscopy (TENSOR-27, BRUKER, Germany) was used to monitor the samples’ surface functional groups (range from 500 to 4000 cm^−1^). The porous texture was investigated with N_2_ adsorption–desorption isotherms at − 196 °C and fitted with BET equation (Micromeritic ASAP2460). The O^1s^ and C^1s^ spectrum of sample surface was conducted with X-ray photoelectron spectroscopy (XPS, ESCALab250XI, VG), the spectrum decomposition from XPS was performed using the XPS PEAK 41 program with Lorentzian–Gaussian function after subtraction of a Shirley background. The crystal structure was identified by powder X-ray diffraction (XRD) (Bruker D8 Advance X-ray diffractometer, D/max 2500, Rigaku, Japan) with Cu Kα radiation (*λ* = 0.15406 nm), the operating voltage and electric current were 40 KV and 40 mA.

### Removal of MB

Typically, a certain amount of prepared hydrochar was added into a sealed wide-mouth bottle with 75 mL MB solution and then placed into a constant temperature shaker (CHA-AB, China) to investigate hydrochar types, initial MB concentration, temperature, pH value and reuse. A UV–VIS spectrophotometer (TU-1810, PERSEE, China) was used to determine MB concentration at different time intervals by withdrawing the aqueous samples for measurement at 664 nm wavelength with standard curve method (Vahdati-Khajeh et al. 2019) (see Additional file 1: Fig. S1). The uptake *Q*_*t*_ (mg/g) and MB removal rate *R* (%) were determined as:

$$Q_{t} = \frac{{\left( {C_{0} - C_{t} } \right)V}}{W},\;R \left( \% \right) = \frac{{\left( {C_{0} - C_{t} } \right)100}}{{C_{0} }},$$

where *C*_0_ and *C*_*t*_ (mg/L) are the MB liquid-phase concentrations at the initial time and *t* minutes, respectively. *V* (L) is MB solution volume, and *W* (g) is the adsorbent weight.

## Results and discussion

### Characterization of samples

The elemental compositions (wt%) of CS hydrochars, glucose-derived HTC and cellulose-derived HTC were analyzed and atomic ratios of oxygen to carbon (O/C) and hydrogen to carbon (H/C) are calculated as shown in Table 1.

**Table 1:** Elemental composition of CS, glucose and cellulose-derived hydrochars

Samples	Elemental composition (wt%)				Atomic ratio (at.)	
	C	H	O	N	O/C	H/C
CS-HTC	65.26	4.96	21.50	1.70	0.247	0.912
CS-H_2_SO_4_-HTC	63.25	4.45	23.60	0.99	0.280	0.844
CS-NaOH-HTC	59.65	4.35	25.19	0.38	0.317	0.875
CS-H_2_SO_4_ + NaOH-HTC	64.32	4.80	24.25	0.36	0.283	0.896
Glucose-HTC	60.26	4.05	26.25	ND	0.327	0.807
Cellulose-HTC	60.68	4.04	26.58	ND	0.329	0.799

*ND* not detected

Besides, nitrogen element contents of CS-H_2_SO_4_-HTC, CS-NaOH-HTC, CS-H_2_SO_4_ + NaOH-HTC are obviously lower than CS-HTC (Table 1, entry 5), which means H_2_SO_4_/NaOH (or both) pretreatment could efficient remove nitrogen in CS biomass raw materials. And NaOH performs better nitrogen removal ability than H_2_SO_4_. Secondly, CS-H_2_SO_4_-HTC, CS-NaOH-HTC and CS-H_2_SO_4_ + NaOH-HTC contain slightly higher O/C ratio and lower H/C ratio. It is generally accepted that decarboxylation, dehydration, condensation polymerization, hydrolysis, and aromatization are the main carbonaceous material generation pathway during HTC process (Khan et al. 2019; Sevilla and Fuertes 2009; Tu et al. 2019), dehydration action could reduce H content. Acid/base (or both) could selectivity remove hemicelluloses and lignin from CS that leads to high cellulose rich in pretreated CS. O-rich cellulose further enhances O content and O/C atomic ratio in hydrochars (Li et al. 2020a; Rusanen et al. 2019; Smith et al. 2016; Sun et al. 2020; Xu et al. 2019; Zhang et al. 2020a).

**Fig. 3:**
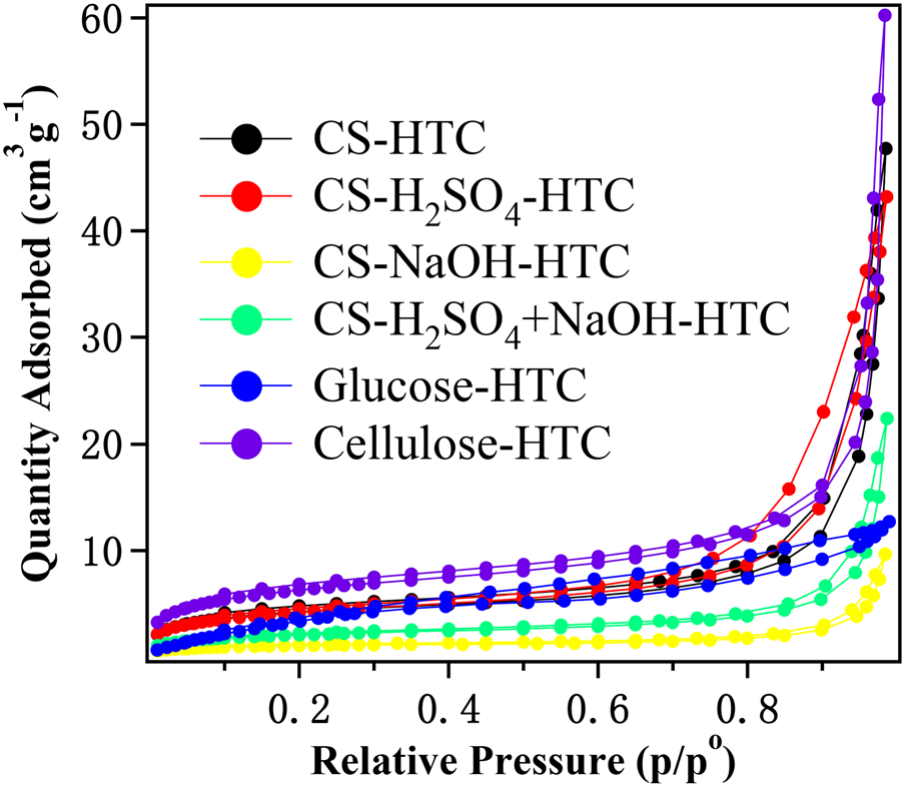
N_2_ adsorption–desorption isotherms of prepared hydrochars

The information of BET surface area, pore volume (by BJH adsorption cumulative) and pore size (by BJH desorption date) of CS, glucose and cellulose-derived hydrochars is depicted in Fig. 3 and Table 2.

**Table 2:** BET surface area, pore volume and pore size of CS, glucose and cellulose-derived hydrochars

Samples	*S*_BET_ (m^2^/g)	Pore volume (cm^3^/g)	Pore size (Å)
CS-HTC	14.51	0.072	263.547
CS-H_2_SO_4_-HTC	14.31	0.065	190.845
CS-NaOH-HTC	3.75	0.014	263.104
CS-H_2_SO_4_ + NaOH-HTC	7.24	0.034	255.788
Glucose-HTC	15.76	0.018	44.867
Cellulose-HTC	22.30	0.090	240.695

As shown in Fig. 3, the isotherm obtained for all samples could be classified as type I and type II isotherms (Román et al. 2012). The curves showed an upward trend near the relative pressure of approximately 0.9, which indicates that the obtained hydrochars contain virtually no framework of confined pores (Adebisi et al. 2016). Information from Table 2 demonstrates the CS-derived hydrochars have poor porosity and low surface area. Cellulose-HTC possessed the highest BET specific surface area (22.3 m^2^/g) as well as the highest pore volume (0.090 cm^3^/g), which implies cellulose plays important role in hydrochar textural structure. Acid/base pretreatment could remove hemicelluloses and lignin (including acid-soluble and base-soluble lignin) that leave much looser tissue structure in plant cell wall (Isikgor and Becer 2015; Yu et al. 2019), however their derived hydrochars do not exhibit high BET surface and pore volume as expected. This phenomenon was also attributed to the fact that lignin improves microspheres, in which pores and gaps are blocked, and more crack structure is exposed (Xiao et al. 2018). As discussed in Fig. 2, NaOH could degrade lignin in CS cell wall, leading low lignin content in CS before HTC process. Thus, it is understandable that CS-H_2_SO_4_ + NaOH-HTC and CS-NaOH-HTC possess lower BET (7.24 and 3.75 m^2^/g, respectively) than CS-H_2_SO_4_-HTC (14.31 m^2^/g). CS-NaOH-HTC behaves the lowest BET surface, it is speculated the hemicellulose residue may generate more complex products during HTC process that block the channel and the pores. Surely more researches and details are expected to help understand HTC process of biomass feedstock.

A typical CS elemental contents for C, H, O and N are 43.22–46.43%, 5.13–6.31%, 38.79–44.98% and 0.68–1.02%, respectively. BET surface area and pore volume are 1.5 m^2^/g, 0.0071 cm^3^/g as reported (Fu et al. 2012; Lu et al. 2021; Zhang et al. 2018a; Zhao et al. 2018). This indicates the HTC process could enhance C content and improve the surface exposure, which are important for dye adsorption.

The feedstock plant tissue and composition play as crucial parameters in the formation of hydrochar. But feedstock constitution effect on hydrochar structure is complex, it is thus hard to distinguish the identified action of a certain component in cell wall during the HTC process, especially the coexistence of other minor compositions (tannin and pectin, for examples) (Nizamuddin et al. 2017; Zhuang et al. 2019). Therefore, it remains to be elucidated in more detail by in situ detection technology.

**Fig. 4:**
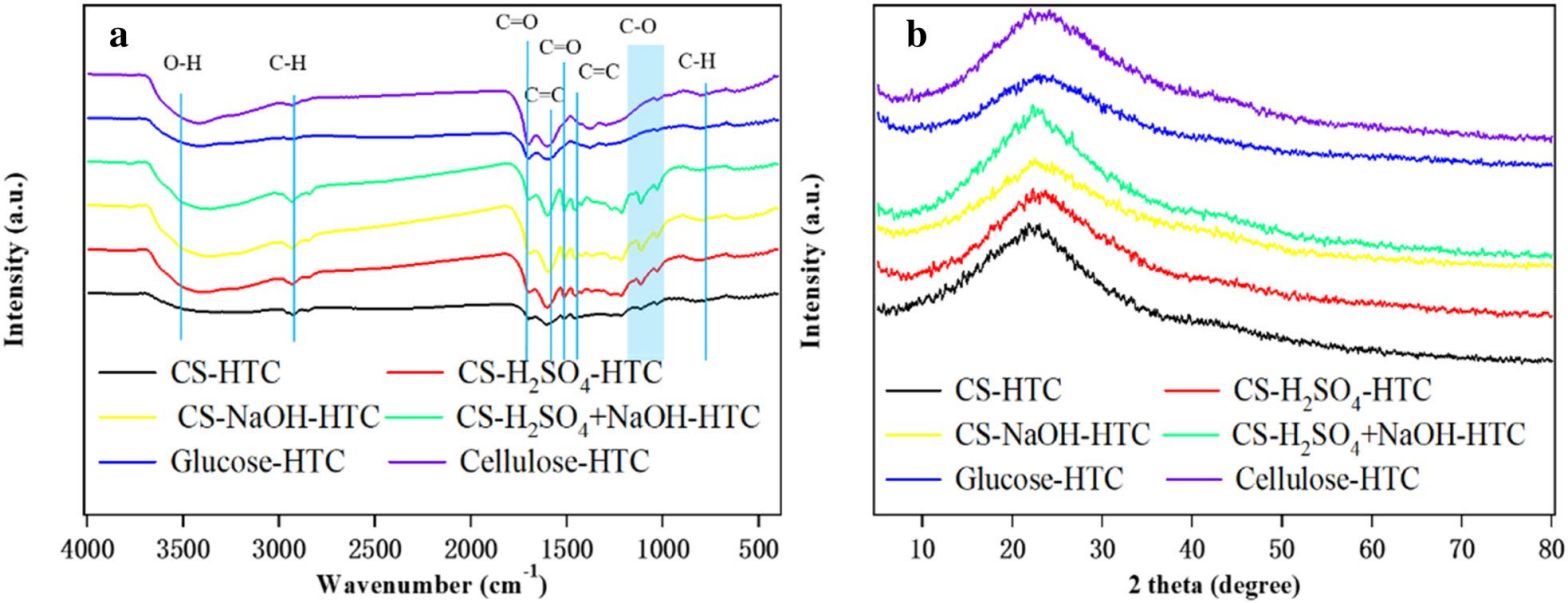
FTIR spectra (**a**) and XRD patterns (**b**) of CS hydeochars, glucose and cellulose-derived hydrochars

FTIR measurement and powder XRD analysis were carried out to get the information of hydrochar surface functional groups and crystal structure and results are presented in Fig. 4.

As observed, the FTIR spectra of CS-HTC, CS-H_2_SO_4_-HTC, CS-NaOH-HTC and CS-H_2_SO_4_ + NaOH-HTC appear to be similar but are different in the peak intensity. The broad FTIR bands at 3300–3500 cm^−1^ are attributed to O–H stretching vibrations in hydroxyl or carboxyl groups (Liu et al. 2019). The band ranges from 2850 to 2950 cm^−1^ is assigned to C–H stretching modes of aromatic and aliphatic. The presence of aromatic is verified by the bands at 1620 cm^−1^ and 1520 cm^−1^, which are related to C=C vibrations. The bands at 1710 cm^−1^ correspond to carboxyl vibrations (C=O). Bands at 1460 and 1440 cm^−1^ are due to the stretching in cyclic amide (C=O) and aromatic skeletal and ester (C=C). The bands at 1000–1250 cm^−1^ are assigned to C–O stretching vibrations in hydroxyl, ester or ether and O–H bending vibrations. The band at 870 cm^−1^ is ascribed to aromatic C–H out-of-plane bending vibrations (Figueiredo et al. 1999)*.* The intensity decreases at the bands of 1620 cm^−1^ for CS-HTC, which suggests a lower degree of graphitization (Buapeth et al. 2019)*.* The higher intensity of bands at 1000–1250 cm^−1^ in CS-H_2_SO_4_-HTC, CS-NaOH-HTC and CS-H_2_SO_4_ + NaOH-HTC means more oxygen-containing groups are formed during the HTC process because of the pretreatment with acid and base. After acid and base pretreatment, the hemicelluloses and lignin are expected to be removed and cellulose-rich materials are obtained, whereas the FTIR spectra of CS-H_2_SO_4_ + NaOH-HTC and Cellulose-HTC are very different. Especially for the range from 1400 to 1800 cm^−1^, the CS-derived hydrochars exhibit devious peaks, indicating that their surface has multiple functional groups. According to literature (Fu et al. 2012), CS raw materials contain a number of atomic groupings and structures, such as hydroxyl, carbonyl, ether group, C–H bond, olefinic C–H and aromatic C=C bond. Natural resistance of plant tissues, lower BET surface area, along with small pore volume, those functional groups are blocked. The HTC process accelerates the new chemical groups formation on the surface of CS hydrochars.

The XRD patterns of HTC hydrochar derived from CS, glucose and cellulose are shown in Fig. 4b. XRD of native CS exhibited diffraction peaks near 15–16°, 22.5°, and 35° (2*θ*), originating from cellulose within CS cell wall (Wang et al. 2016), while in the CS-HTC hydrochars, because of the hydrolysis reaction and further conversion of cellulose under HTC temperature, the peaks at 15–16° and 35° (2*θ*) have been replaced with a broad diffraction peak located at 22°, which corresponds to the (002) diffraction pattern of amorphous carbon (Wang et al. 2016). As summarized in reported literature (Salimi et al. 2017), the HTC crystal structure relies heavily upon HTC conditions, especially the temperature and residue time. The obtained hydrochars are all amorphous, which is intelligible under our HTC operation conditions (220 °C and 6 h).

**Fig. 5:**
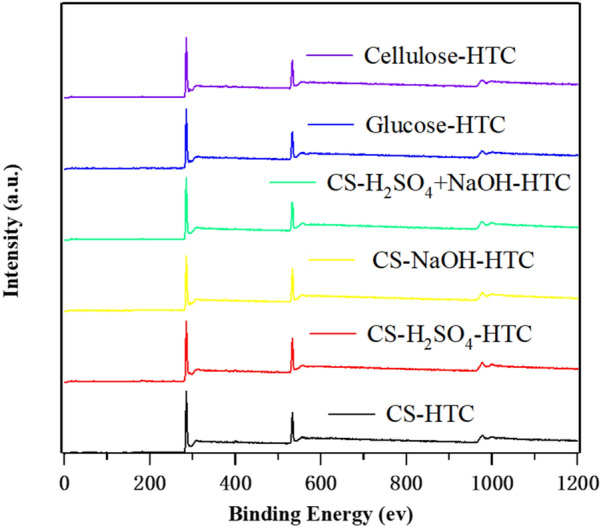
XPS full-spectra of CS hydrochars, glucose and cellulose-derived hydrochars

XPS full-spectra of CS, glucose and cellulose-derived HTC samples are shown in Fig. 5. The detailed fitting is supplied in Additional file 1. Similar to the virgin CS as reported in literature (Ren et al. 2015), the O^1s^ XPS spectral reveals that abundant O-contained functional groups are on the hydrochar surface, especially the carboxy group (–COOH). Those O-contained groups are crucial for carbon materials adsorption ability (Chen et al. 2017).

### Comparison of different hydrochar samples

**Fig. 6:**
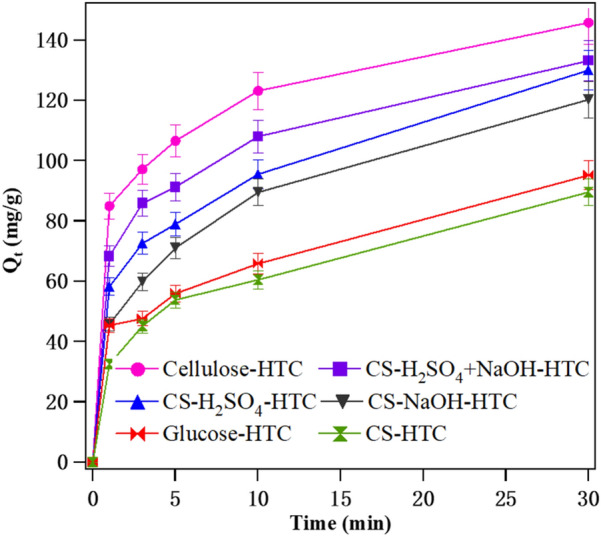
MB uptake (*Q*_t_) versus time of CS hydrochars, glucose and cellulose-derived hydrochars. Adsorption conditions: MB initial concentration 100 mg/L, MB water solution volume 75 mL with a pH value of 7, adsorbent dosage is 50 mg, 30 °C and 120 rpm

The comparison of original CS, pretreated CS with acid/base (or both), glucose and cellulose-derived hydrochar is performed by measuring their MB adsorption rate; the result is shown in Fig. 6.

Cellulose-HTC presents the fastest MB adsorption rate and maximum adsorption capacity within test time. Glucose-HTC and CS-HTC show almost the same MB adsorption rate and adsorption capacity, which have the worst performances. Pretreatments with acid, base or both could significantly enhance CS-derived hydrochar MB adsorption ability. And the effects of pretreatment could be ordered in the sequence of H_2_SO_4_ + NaOH, NaOH and H_2_SO_4_.

Cellulose possesses a rod-like structure with the aid of flat bond conformation of the glucose residue. During cellulose-HTC process, the reactions of cellulose into glucose by hydrolysis and glucose into 5-hydroxymethylfurfural, levulinic acid by dehydration firstly take place (Liu et al. 2019)*.* Experimental results show the cellulose-derived hydrochar performed better than glucose-derived hydrochar. Thus, we think the biomass raw material textural structure plays an important role on their hydrochar characterizations. Compared to glucose, cellulose is of chains that closely linked each other to form fibrous network structure because of the intermolecular hydrogen bonding (Medronho and Lindman 2015). Those fibrous network structures are beneficial to hydrochar pore volume and pore size (0.090 m^3^/g, 240.695 Å for Cellulose-HTC and 0.018 m^3^/g, 44.867 Å for glucose-HTC as listed in Table 2) (Titirici et al. 2007), as well as to hydrochar MB adsorption capacity.

Acid/base or both pretreatments could remove hemicelluloses, lignin, and some other acid/base-soluble or N-containing components in biomass raw material (Somsesta et al. 2020; Zhang et al. 2019). Thus, the structures of pretreated CS are similar to cellulose, which contains weakly connected plant tissues. Therefore, it is easy to understand CS-H_2_SO_4_, CS-NaOH-HTC and CS-H_2_SO_4_ + NaOH-HTC have higher MB adsorption rate and adsorption capacity than CS-HTC. However, among the three pretreated CS-derived hydrochars, CS-H_2_SO_4_ + NaOH-HTC performed as the best adsorbent, which is confused. Pore volume, pore size, BET surface, along with surface functional groups are crucial for carbon materials during dye removal (Jain et al. 2016). Whereas there is no absolute advantage for the above-mentioned factors in CS-H_2_SO_4_ + NaOH-HTC according to Table 2. Acid treatment could remove most hemicelluloses and acid-soluble lignin in CS, base pretreatment removes most lignin, both acid and base pretreatments degrade hemicelluloses and lignin that leave cellulose-rich weakly connected plant tissues, while textural information does not exhibit a certain regularity, indicating the interaction among the three major components (cellulose, hemicelluloses and lignin) in CS during HTC is rather complex. Especially the CS adopted in this study has not gone through treatment with benzene extraction or alcohol extraction after harvesting. Thus, the influence of the existence of lower content compositions (tannic and pectin and ash content, for example) (Jiang et al. 2020) on the hydrochar structure and their adsorption ability are undefined. Another pheromone observed from the elemental analysis is that the N-atom content dropped significantly in pretreated CS hydrochars (Table 1, entry 5). But according to the literature, the N-rich carbon is of high adsorption capacity (Vahdati-Khajeh et al. 2019). This result further illustrates the relationship between raw material plant tissue and their derived hydrochar structural adsorption ability is complex. Synergistic effect of the components within CS structure determines the CS-derived hydrochar MB adsorption capacity. Although some deeper understandings are undefined, this study develops a simple “acid + base” pretreatment method for the directly collected CS (without any extraction), that could improve its dye adsorption capacity. The absence of an extraction process is of great significance on the biomass raw material’s direct utilization. Meanwhile, this study demonstrates that some pretreatment methods are helpful for high-valued biomass-based materials.

Surely, limitations of homogenous acid and base pretreatment of CS are obvious: the generation of polluted water with acid and base residue, along with serious corrosion to the pretreatment equipment. As an alternative way, some solid acid and base may be more suitable for the waste biomass pretreatment for the production of high-quality functional materials.

### Effect of initial MB concentration, temperature, pH and reusability

**Fig. 7:**
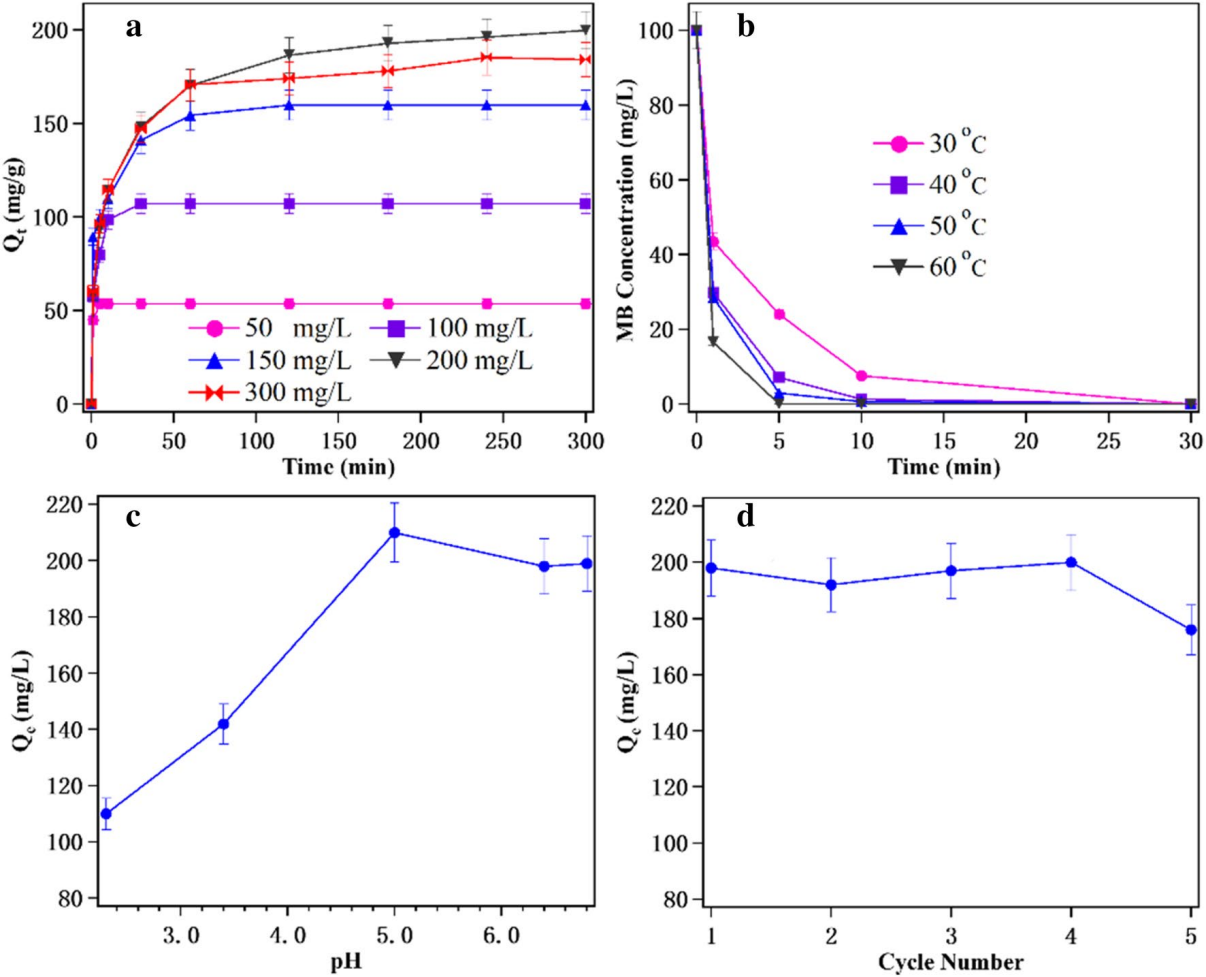
Effect of initial concentration (**a**), temperature (**b**), solution pH (**b**) and reusability on remove of MB onto CS-H_2_SO4 + NaOH-HTC. Adsorption conditions: **a** MB water solution volume is 75 mL with a pH value of 7, adsorbent dosage is 70 mg, 30 °C; **b** MB water solution volume 75 mL with a concentration of 100 mg/L with a pH value of 7, adsorbent dosage is 70 mg; **c** MB water solution volume 75 mL with a concentration of 300 mg/L, adsorbent dosage is 70 mg, 30 °C and 120 min; **d** MB water solution volume 75 mL with a concentration of 300 mg/L, adsorbent dosage is 70 mg, 30 °C and 120 min. The shake speed for each batch experiment is 120 rpm

To gain further insight into the CS hydrochar MB dye adsorption capacity, taking CS-H_2_SO_4_ + NaOH-HTC as the adsorbent, the parameters like initial MB concentration, temperature, pH value and reusability on adsorption were studied and results are shown in Fig. 7.

MB concentrations were investigated to evaluate the adsorption of MB dyes onto CS-H_2_SO_4_ + NaOH-HTC, shown in Fig. 7a. The adsorption reached equilibrium at low MB concentrations of 50 and 100 mg/L within 10 min and subsequently remained constant. Higher MB concentrations required a longer time (25–120 min) to reach equilibrium. Eventually, the needed contact time for MB initial concentration of 300 mg/L reached up to 120 min to reach equilibrium under fixed adsorbent dosage. This observation was in agreement with the results reported by some other research groups (Islam et al. 2015; Li et al. 2011) and can be explained by the fact that molecule adsorptive competition toward the active adsorption sites of CS-H_2_SO_4_ + NaOH-HTC surface is intensified because of the increasing initial MB, which leads to an enhanced equilibrium time (Chen et al. 2010). The maximum MB adsorption capacity of MB onto CS-H_2_SO_4_ + NaOH-HTC is 198.0 ± 9.8 mg/g, which is higher than CS-H_2_SO_4_, CS-NaOH and CS-H_2_SO_4_ + NaOH (maximum MB adsorption capacity are 120.1 ± 5.6, 123.4 ± 4.3, 126.5 ± 7.2 and 135.7 ± 6.2 mg/g, respectively). It is thought the HTC process make more C content and expose more surface as discussed above. The MB removal rate *R* (%) for CS-H_2_SO_4_ + NaOH-HTC is calculated by the equation *R* (%) = $\frac{{100Q_{t} W}}{{V*C_{0} }}$, the results reveal that for initial MB concentration of 50, 100, 150, 200 and 300 mg/L, the MB removal rate are 100%, 100%, 99.4%, 93.2% and 57.3%, respectively, which exhibits excellent adsorption capacity under lower MB concentration.

The effect of temperature on MB removal was also studied. Result reveals the adsorption rate of MB onto CS-H_2_SO_4_ + NaOH-HTC is proportional to temperature. As known, the whole adsorption process of aqueous molecules onto adsorbent includes two steps: (1) MB molecules transfer from solution to the adsorbent near surface; and (2) the adsorption from near surface to the adsorbent surface (Gadekar and Ahammed 2019). The thermodynamic analyses have been done and the details can be seen in Additional file 1. The fitted ∆*H* = 23.76 kJ/mol, which is below 40 kJ/mol, implying that the adsorption of MB by CS-H_2_SO_4_ + NaOH-HTC is a physisorption process (Neolaka et al. 2018; Shu et al. 2018).

pH value of the solution can affect MB adsorption onto CS-H_2_SO_4_ + NaOH-HTC by adjusting surface charges. The capacity of MB adsorption in the presence of different pH values ranging from 2.0 to 7.0 was detected as shown in Fig. 7c. Results indicate that the pH value of solution exerted a significant influence on the MB adsorption onto CS-H_2_SO_4_ + NaOH-HTC. At lower pH, cationic MB resists to combine with acidified adsorbent surface because of the electrostatic repulsive force that weakens mass transfer and leads to a reduction in MB removal percentages (Ghaedi et al. 2016; Zhang et al. 2019). The maximum MB adsorption was achieved at a pH value of 7.0, which can be explained by the fact that the absented positive charge on H_2_SO_4_ + NaOH-HTC surface can enhance MB mass transfer and adsorption. An alkali environment was not investigated in this study because there is an agreement that the competitive MB adsorption onto reactive sites with abundant OH^−^ in solution will lead to the MB adsorption reduced (Ghaedi et al. 2016). In fact, aniline and malachite green oxalate salt were also tested to evaluate their adsorption capacity onto H_2_SO_4_ + NaOH-HTC at the same conditions (adsorbent dosage is 70 mg, 30 °C and 120 min). The maximum adsorption are 19.2 ± 2.0 mg/L and 319 ± 13.2 mg/L for aniline and malachite green oxalate salt, respectively. The low aniline adsorption ability is due to its low solubility in water. And malachite green oxalate salt is cationic that can readily adsorb onto H_2_SO_4_ + NaOH-HTC surface under neutral solution as well as MB.

Recyclability of H_2_SO_4_ + NaOH-HTC was investigated and shown in Fig. 7d. The regeneration of adsorbent was realized by washing with HCl (2 M) solution, ethanol and DI water to remove impurity and adsorbed dye completely (Vahdati-Khajeh et al. 2019). Finally, the regenerated adsorbent was dried at 50 °C and used in the next run. The maximum MB adsorption remained at 176 ± 8.6 mg/L over four adsorption–desorption cycles with a slightly reducing of 198 ± 9.8 mg/L at the first adsorption test, suggesting that HCl and ethanol can be used as good desorption/regeneration agents and the H_2_SO_4_ + NaOH-HTC possesses high stability.

## Conclusion

Conclusively, we investigated the influence of simple acid/base or both pretreatment on agricultural residue biomass cotton stalk (CS) derived hydrothermal carbonization. The chemical structures and properties of the prepared hydrochars are compared by their methylene blue (MB) dye adsorption capacity. Material characterization and MB removal test reveal that selective removal of components within CS with the aid of acid/base (or both) is of benefits to the preparation of high-quality carbonaceous materials by the HTC method. During the MB adsorption onto CS-H_2_SO_4_ + NaOH-HTC, initial MB concentration evaluation reveal that high MB initial concentrations require longer time to reach equilibrium for the cases with fixed adsorbent dosage. Temperature investigation demonstrates the adsorption process is a physisorption process. pH value of solution affects the hydrochar surface electrical charge as well as MB removal ability. Finally, the recyclability test indicates the CS-derived hydrochar could be easily regenerated by simple washing with HCl and ethanol. It is noted that the CS used in this study was without any extraction after harvesting that simplifies the process and of great significance on the direct utilization of waste biomass.

### Supplementary Information

**Additional file 1.** Supporting information.

## Data Availability

All data generated or analyzed during this study are included in this article.

## Abbreviations

CS: Cotton stalk
HTC: Hydrothermal carbonization
FTIR: Fourier transform infrared
XPS: X-ray photoelectron spectroscopy
XRD: X-ray diffraction
MB: Methylene blue
H_2_SO_4_: Sulfuric acid
NaOH: Sodium hydroxide
KOH: Potassium hydroxide
HCl: Hydrochloric acid
