# Supplementary material for: Cotton stalk-derived hydrothermal carbon for methylene blue dye removal: investigation of the raw material plant tissues
Source: Bioresour Bioprocess. 2021 Jan 30;8(1):10. doi: 10.1186/s40643-021-00364-8 (PMC10992739; doi:10.1186/s40643-021-00364-8)
Supplement: Supplementary file 1 — Additional file 1. Supporting information. [file 40643_2021_364_MOESM1_ESM.docx]

Cotton stalk derived hydrothermal carbon for methylene blue dye removal: investigation of the raw material plant tissues

Libo Zhang^1,*^, Junyan Tan^2^, Gangying Xing^3^, Xintong Dou^3^, Xuqiang Guo^1^

^1^State Key Laboratory of Heavy Oil Processing, College of Engineering, China University of Petroleum-Beijing at Karamay, Karamay 834000, People’s Republic of China

^2^Shenzhen College of International Education, Shenzhen 518048, People’s Republic of China

^3^State Key Laboratory of Heavy Oil Processing, China University of Petroleum, Beijing 102249, People’s Republic of China

^*^Corresponding authors.

Libo Zhang, Email: zhanglibo@cupk.edu.cn Tel: +86-0990-6633346,

State Key Laboratory of Heavy Oil Processing, College of Engineering, China University of Petroleum-Beijing at Karamay, Karamay 834000, People’s Republic of China

**Figure S1. MB standard curve measurement with UV-VIS at 664 nm wavelength.**

**C1S**


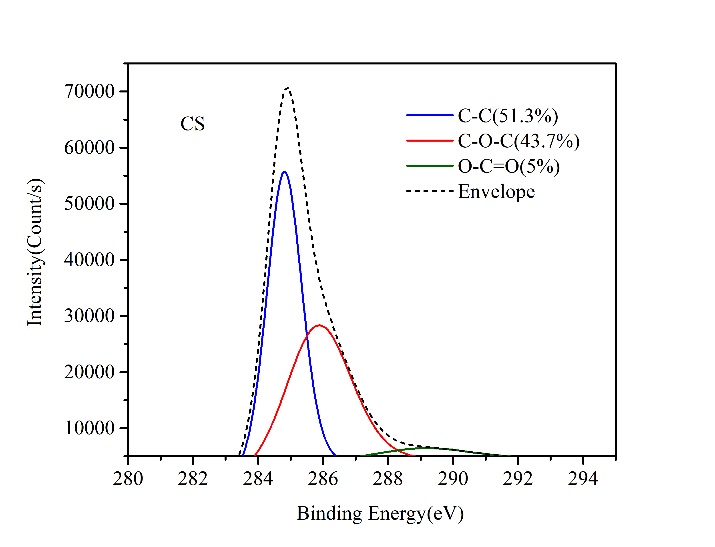

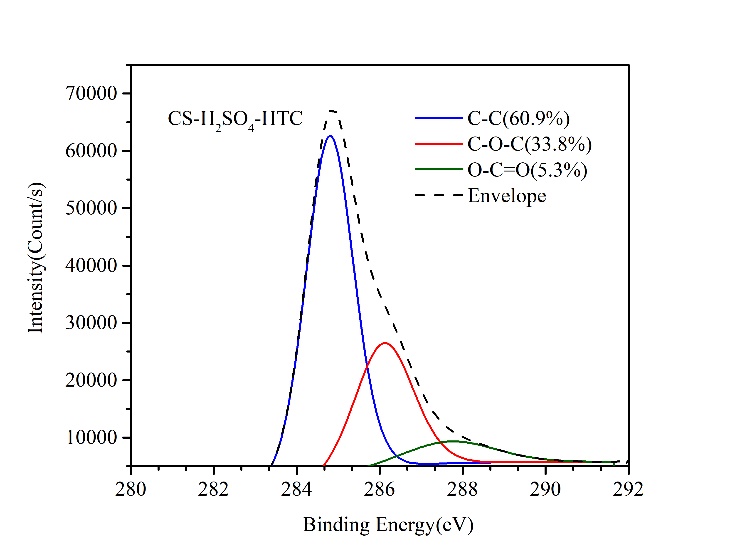


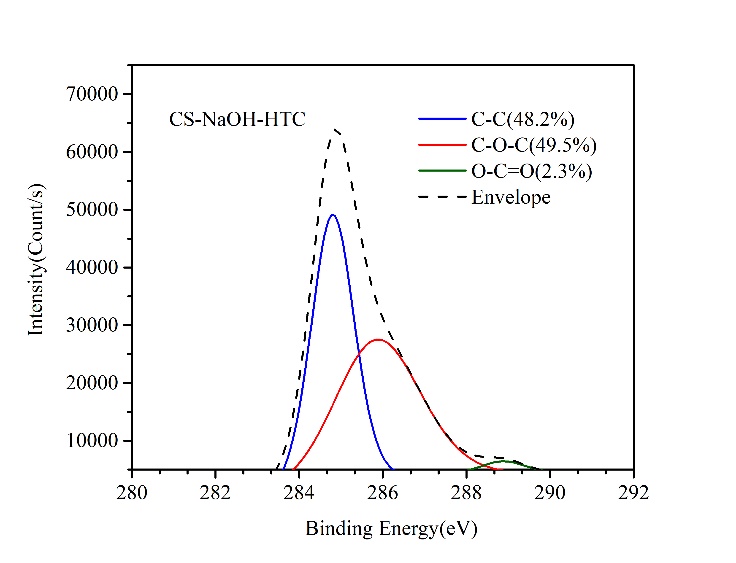

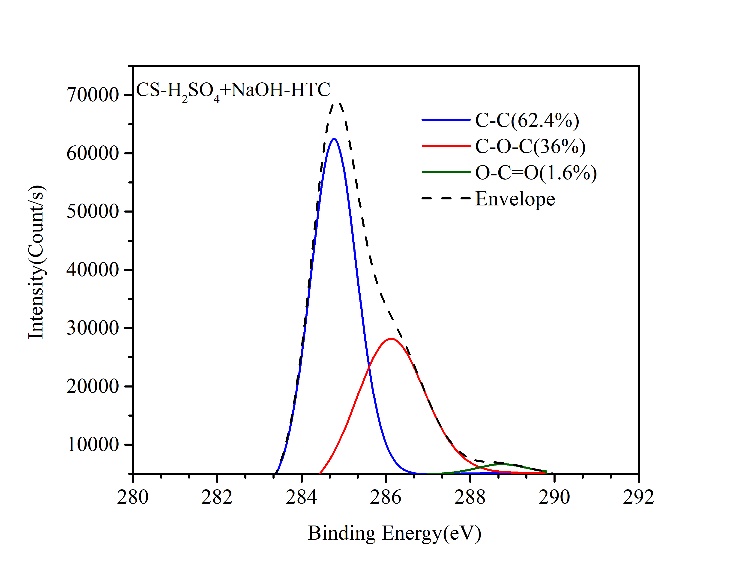


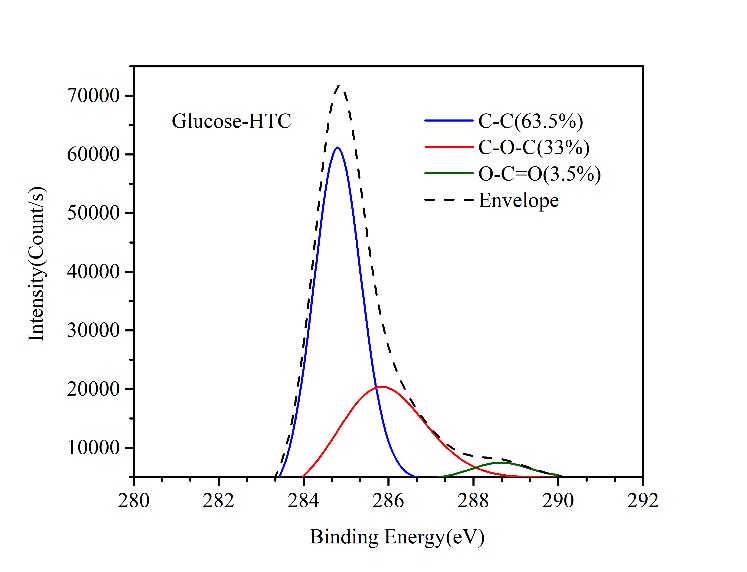

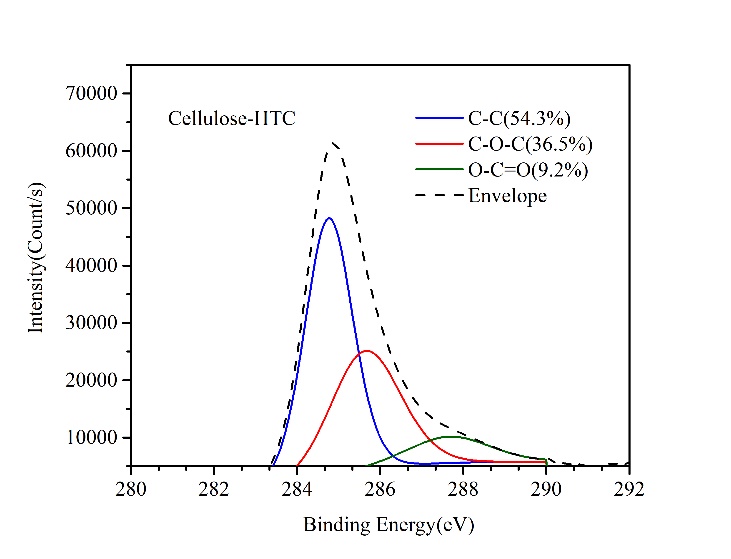


C and O XPS spectral of CS derived

**O1S**


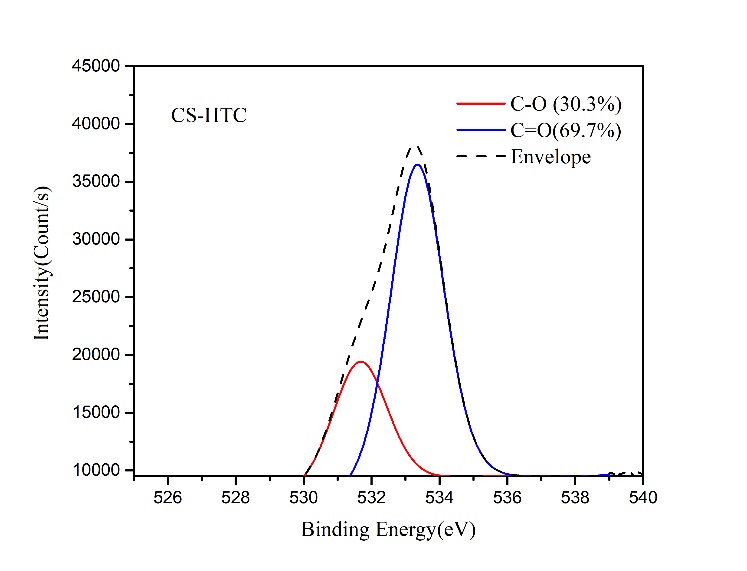

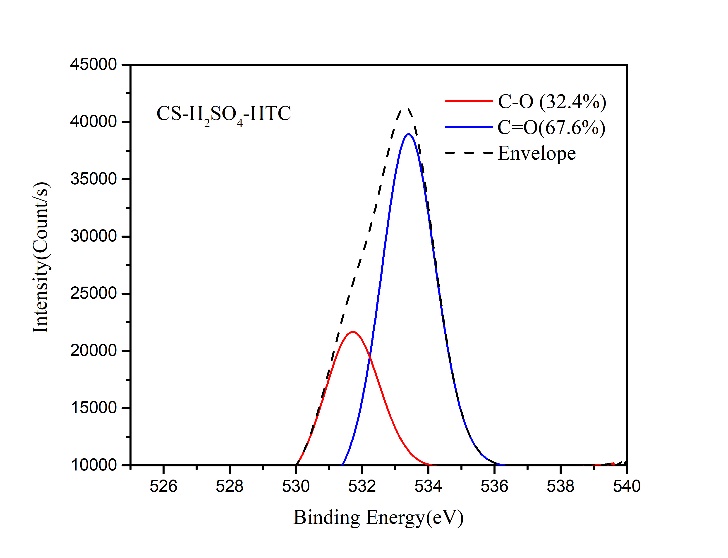


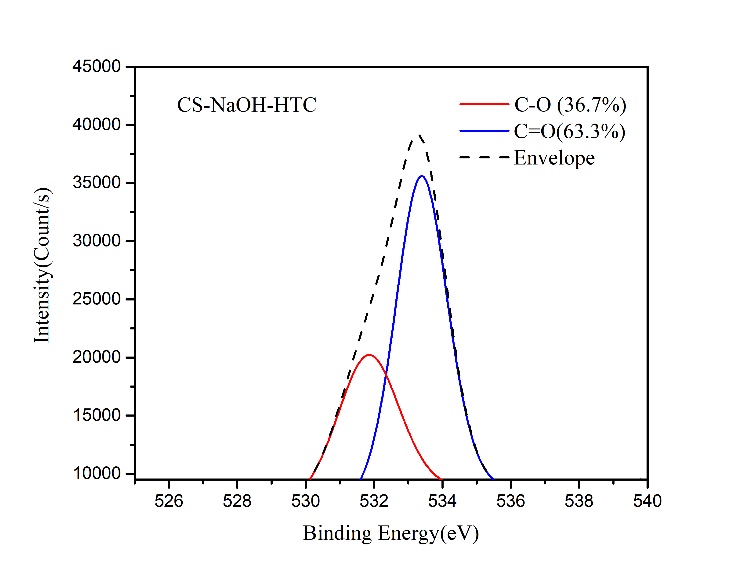

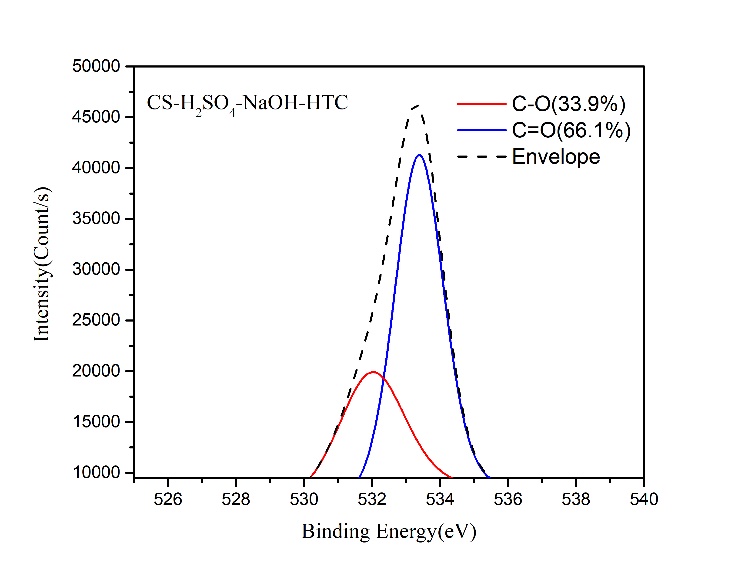


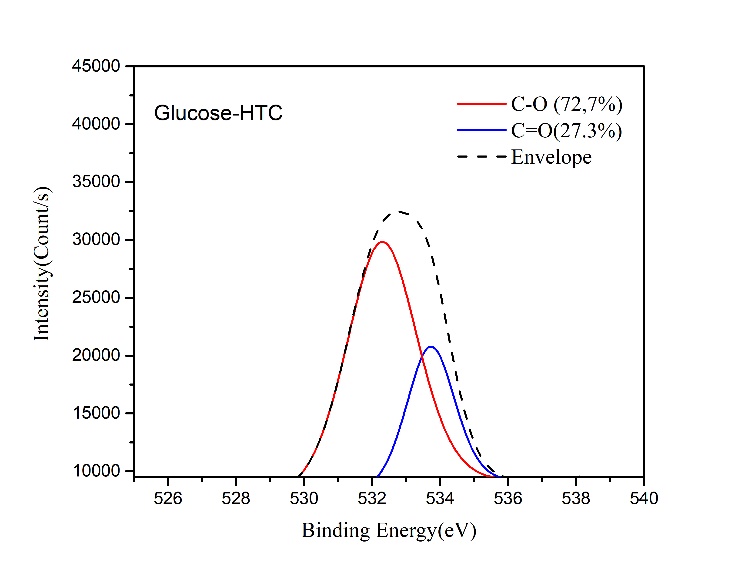

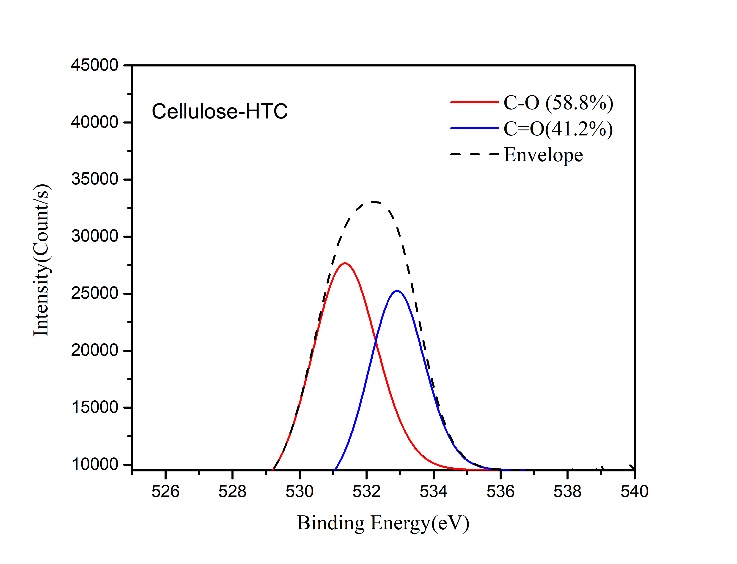


∆H fitting process:

The ∆H is fitted by the equation as below:

$\ln\left( \mathrm{Kc} \right)=\frac{\Delta S}{R}-\frac{\Delta H}{RT}$ (1)

$K_{C}=\frac{C_{s}}{C_{e}}$ (2)

$\Delta G=-RT\ln K_{c}$ (3)

$\Delta G=\Delta H-T\Delta S$ (4)

Where:

Kc: Langmuir equilibrium constant (L/mol),

Cs: Concentrations of MB adsorbed on adsorbent

Ce: Concentrations of MB in solution.

R: Universal gas constant (8.314 J/(K*mol))

T: Absolute temperature in Kelvin (K).

**Table S1.** Basic data of hydrothermal carbon adsorption methylene blue

| T(K) | 303 | 313 | 323 | 333 |
| --- | --- | --- | --- | --- |
| 1/T | 0.003300 | 0.003194 | 0.003095 | 0.003003 |
| $C_{0}$(mg/L) | 300 | 300 | 300 | 300 |
| $C_{s}$(mg/L) | 196.83 | 213.21 | 233.77 | 243.90 |
| $C_{e}$(mg/L) | 103.17 | 86.79 | 66.23 | 56.10 |
| $\mathrm{Kc}$ | 1.908 | 2.457 | 3.530 | 4.348 |
| $\ln\left( \mathrm{Kc} \right)$ | 0.6464 | 0.8989 | 1.2613 | 1.4698 |

**Table S2.** Thermodynamic fitting data

| Intercept ($\frac{\Delta S}{R}$) | Slope ($-\frac{\Delta H}{R}$) | R^2^ |
| --- | --- | --- |
| 10.07 | -2858.78 | 0.98689 |

According to Eq(1), the $\Delta H$ and $\Delta S$ values could be calculated from the slope and intercept of the linear plot of $\ln\left( \mathrm{Kc} \right)$ versus 1/*T*, to be $\Delta H$ = 23.76kJ/mol and ΔS = 0.083 J/molK . ΔG values at 313.15, 323.15, 333.15, and 343.15 K were calculated to be −1.389, −2.219, −3.049 and −3.879kJ/mol, respectively.

**
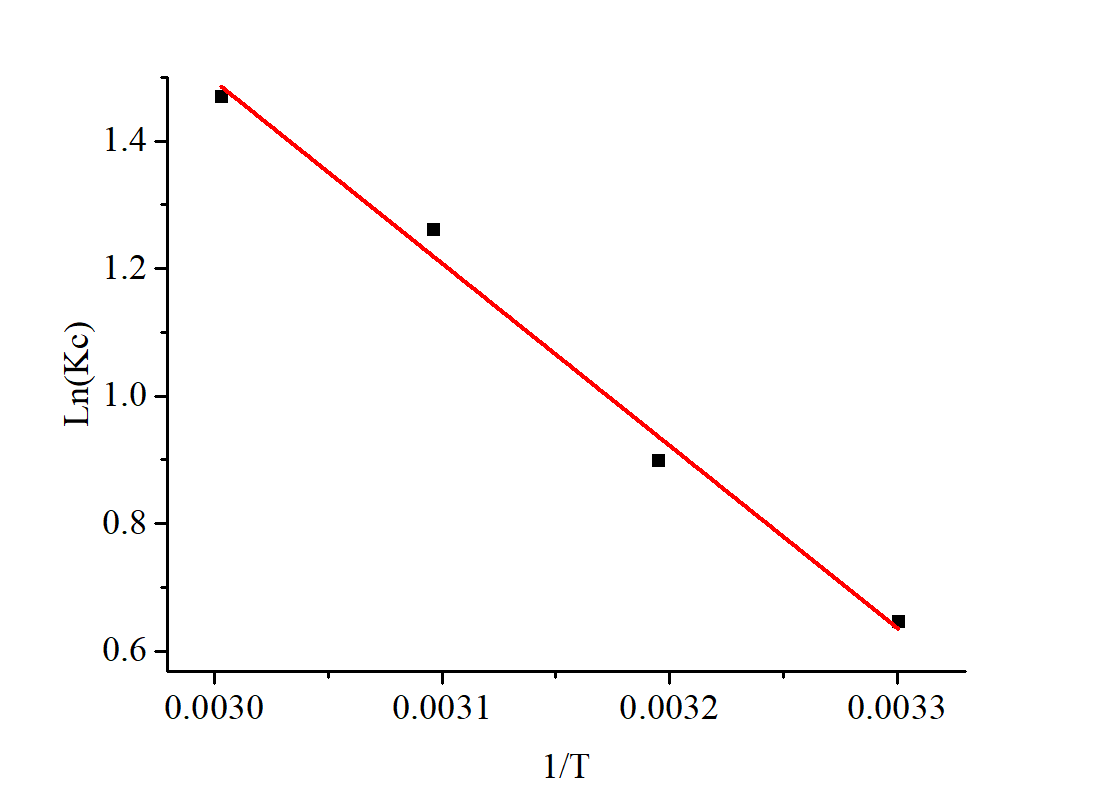
**
